# Multifocal osteoclast-rich tumour in Paget bone disease and conventional giant cell tumour, two genetically distinct entities? Sequencing from a single case

**DOI:** 10.1007/s00256-023-04369-6

**Published:** 2023-06-13

**Authors:** Simon Haefliger, Judith Bubbear, Christropher Davies, Lucia Cottone, Fernanda Amary, Roberto Tirabosco, Isidro Cortes-Ciriano, Paul O’Donnell, Adrienne M Flanagan

**Affiliations:** 1 Present Address: Research Department of Pathology, University College London, UCL Cancer Institute, WC1E 6BT, London, UK; 2 Cellular and Molecular Pathology, Royal National Orthopaedic Hospital, Greater London, Stanmore, UK; 3 Centre for Metabolic Bone Disease, Royal National Orthopaedic Hospital, Greater London, Stanmore, UK; 4 European Molecular Biology Laboratory, European Bioinformatics Institute, Hinxton, Cambridge, UK; 5 Department of Radiology, Royal National Orthopaedic Hospital, Greater London, Stanmore, UK

**Keywords:** Paget disease of bone, Giant cell tumour of bone, Osteoclast-rich tumour, Genetics, Whole exome sequencing

## Abstract

Paget disease of bone is a metabolic disorder with a strong genetic component, characterised by pronounced disorganised bone remodelling. Complications of this disease include an increased risk of developing bone neoplasms. Here, we describe the case of a 60-year-old Italian patient with Paget disease of bone, presenting with an osteoclast-rich tumour. Our analysis of this entity, based on the clinical, morphological and genetic data (whole exome sequencing), suggests that osteoclast-rich lesions in Paget disease of bone are genetically distinct from classical giant cell tumour of bone. We discuss the importance of differentiating these osteoclast-rich lesions.

## Introduction

Paget disease of bone (PDB) is a metabolic disorder with a strong genetic component, characterised by pronounced disorganised bone remodelling [1]. Complications of PDB include an increased risk of developing bone neoplasms, particularly osteosarcoma but not conventional giant cell tumour of bone (cGCTB) as defined by *H3F3A* mutations [2]. Recently, GCT-like lesions have been reported in patients affected by PDB with germline mutations in the zinc finger protein 687 (*ZNF687*) and *PFN1* genes in Italian and Chinese populations, respectively [3, 4]. Germline alterations in S*QSTM1* (up to 50%) and much less frequently in *TNFRSF11A, CSF1, VCP* and *FKBP5* are also implicated in PBD, but these have not been associated with cGCTB [1].

## Case report

**Fig. 1:**
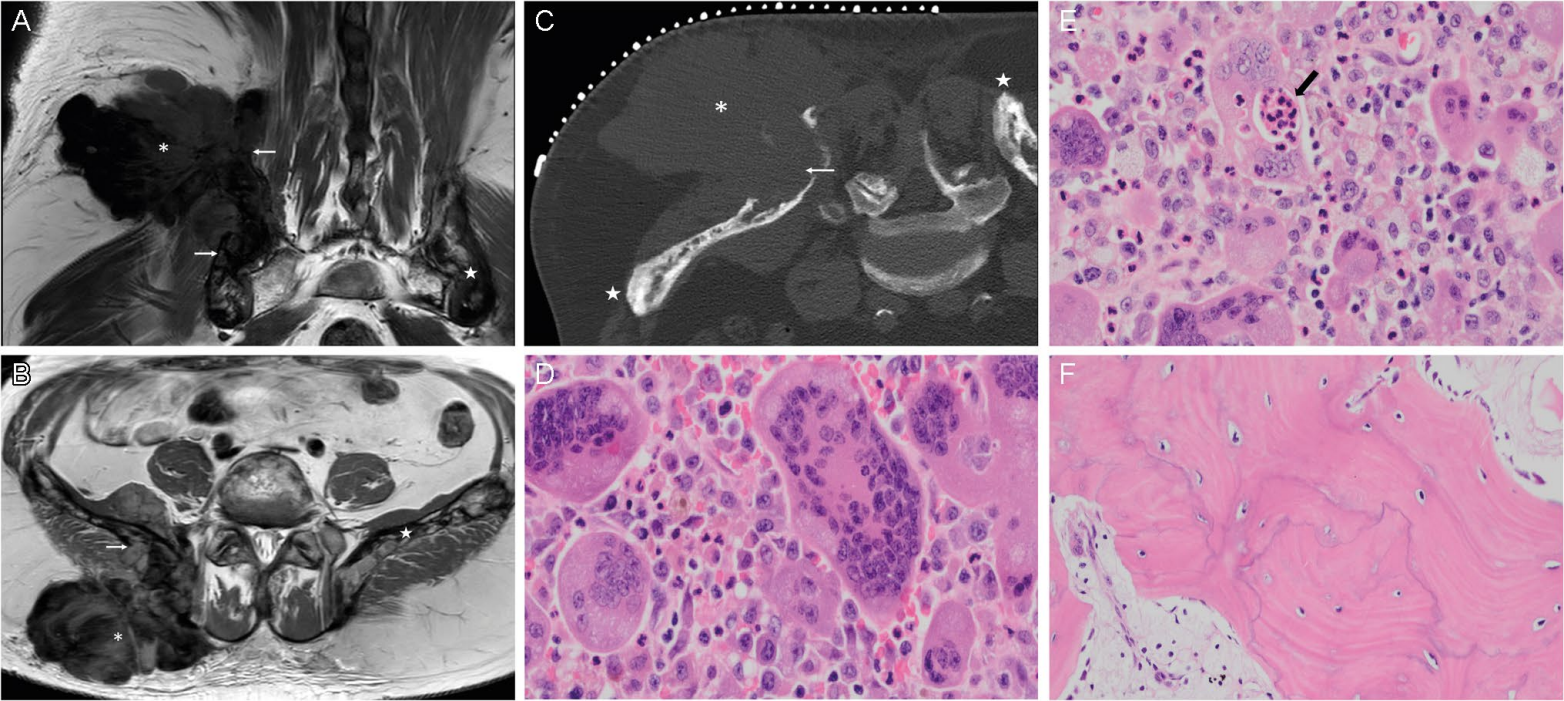
Radiological and histological features of the pelvic lesion. Magnetic resonance coronal T1-weighted and axial proton density images (**A**, **B**) showing a hypointense extraosseous mass (*) arising from the posterior right ilium (arrows). Diffuse changes of PDB can be seen in the right and left (star) iliac bones (**B**). Prone axial CT at the time of biopsy (**C**) showing a non-mineralised mass (*), lytic destruction of the right posterior ilium (arrow) and typical Paget disease of the iliac bones (star). Representative histology of the pelvic lesion showing a cellular lesion with very large OGCs; the accompanying mononuclear component was discohesive with an absence of fibrous matrix (**D**). Numerous OGCs showed emperipolesis (arrow) of neutrophil polymorphs (**E**). The lamellar bone exhibited a mosaic pattern, consistent with PDB (**F**)

A 60-year-old male patient originally from the Campania region of Southern Italy presented with a three-month history of low back pain and a firm mass in the right gluteal region. Seven years previously following a fall, radiographs of the pelvis had shown characteristic appearances of Paget disease affecting the right ilium, sacrum and L4 vertebra. MRI demonstrated a mass arising from the posterior aspect of the right ilium with a large lobular extraosseous component extending into gluteal muscles and subcutaneous fat, measuring 17 cm in maximum dimension (Fig. 1A,B). The mass was hypointense on all MR sequences. MRI also showed diffuse pelvic Paget disease, and uncomplicated PDB was confirmed in the left humerus, left clavicle, cervical and lumbar spines on whole-body MRI. A biopsy was performed under CT guidance, CT images showing that the mass was not mineralised (Fig. 1C).

Histology from the biopsy and subsequent resection of the tumour showed a cellular osteoclast-rich lesion with features not entirely typical of cGCTB. Specifically, the osteoclast-like giant cells (OGCs) were, in general, larger (Fig. 1D), and the accompanying mononuclear component was discohesive. Emperipolesis with engulfment of neutrophil polymorphs by OGCs was also an unusual feature (Fig. 1E). Mitotic figures and necrosis were not seen. The ilium adjacent to the tumour showed characteristic histological features of PBD (Fig. 1F**)**. Immunostains were negative for *H3F3A/B* G34W, *H3F3* K36M (the molecular hallmarks of cGCTB and chondroblastoma respectively), and a *USP6* rearrangement was not detected by fluorescence in situ hybridisation excluding an aneurysmal bone cyst (not shown). The rare *H3F3A* variants (G34L/V/R/M) were not detected on a DNA next-generation sequencing panel [2].

**Fig. 2:**
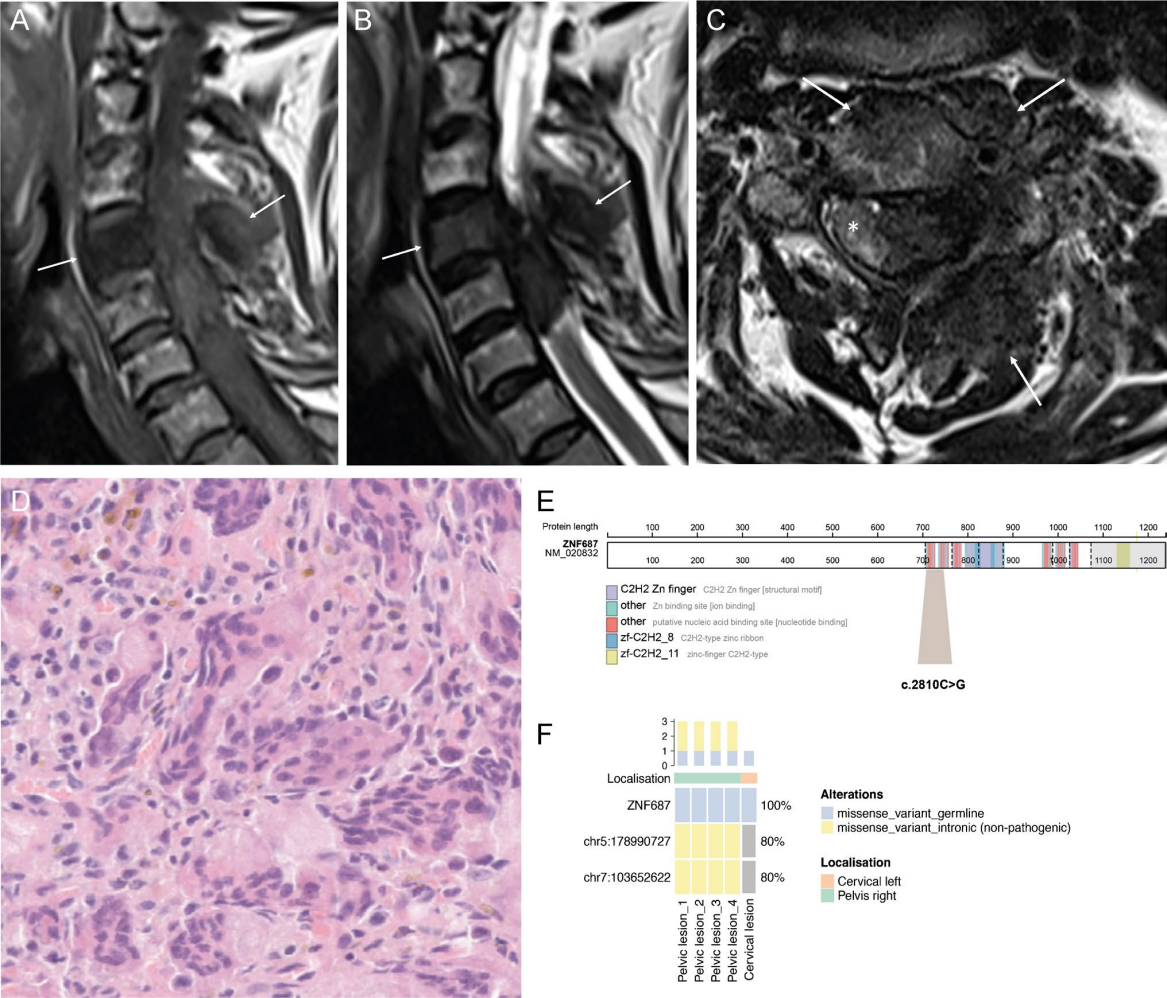
Radiological and histological features of the cervical lesion and sequencing results. Magnetic resonance sagittal T1- and T2- (**A**, **B**) and axial T2-weighted images (**C**) showing hypointense marrow replacement in the C4 body and neural arch (arrows) with an epidural extraosseous mass. The mass displaces the spinal cord (*) to the right. Representative histology of the cervical lesion with less prominent OGCs compared to the pelvic lesion (**D**). In this location, the OGCs were embedded in fibrous tissue and emperipolesis was not identified. Schematic illustration of the ZNF687 protein evidencing the locus with the c.2810C>G amino acid change (p.P937R) (**E**). Oncoprint highlighting the frequency of the detected mutations in the pelvic and cervical lesion (**F**)

Four months after surgery, the patient presented with a six-week history of severe neck/left shoulder pain and left-hand weakness. There was no lower limb weakness, but increased tone was identified on physical examination. MRI revealed marrow replacement in the body and neural arch of the fourth cervical vertebra and an extraosseous mass in the paravertebral soft tissues and epidural space on the left, displacing and compressing the cord to the right (Fig. 2A–C). The vertebral and extraosseous components were hypointense on T2-weighted MR images, the extraosseous component showing isointense T1 signal (Fig. 2A); CT confirmed diffuse changes of PDB in the cervical spine and showed that the mass was not mineralised. This biopsy revealed an osteoclast-rich lesion with histological features more in keeping with a cGCTB: emperipolesis was not a feature, and OGCs were smaller than in the pelvic lesion. The cells were also embedded in a fibrous background, possibly reactive secondary to the vertebral compression (Fig. 2D**)**. Immunostains for *H3F3A/B* G34W, *H3F3* K36M were negative, and a histone mutation was not detected on DNA sequencing, indicating that the lesion did not represent a cGCTB. Surgical decompression was undertaken successfully. As the patient originated from Italy and in the light of the recent description of germline mutations in patients of Italian origin with PDB and GCT-like lesions [3], capillary sequencing of his blood was undertaken and revealed the previously reported hotspot heterozygous *ZNF687* (Exon 6 – p.P937R, c.2810C>G) mutation (Fig. 2E).

To investigate if the large osteoclast-rich tumours in this patient were due to additional somatic genetic alterations, we performed whole exome sequencing to a depth of 250× on five frozen tumour samples including four regions of the pelvic lesion and one region of the cervical mass. Details of the analysis pipeline used can be found in the Supplementary information. Sequencing confirmed the presence of the *ZNF687* mutation (Exon 6 – p.P937R, c.2810C>G) in all samples. No major alterations in copy number were seen, and pathogenic somatic driver alterations were not identified in any of the samples. However, two intronic, non-pathogenic somatic mutations (chr7:103652622 and chr5:178990727) were found in the pelvic but not in the cervical lesion (Fig. 2F).

## Discussion

We report metachronous osteoclast-rich tumours occurring in a patient with polyostotic PDB. The initial clinical and imaging concerns were of PDB-related malignancy or cGCTB, and metastasis was considered when a mass with similar imaging appearances developed in the cervical spine four months after the iliac lesion was treated. Although both lesions were histologically benign, the pelvic lesion did not exhibit the typical morphological features or the hallmark genetic alteration, a mutation in *H3F3A/B* G34, of cGCTB. Furthermore, the cervical lesion shared morphological similarities with cGCTB, but, similar to the pelvic lesion, did not harbour an *H3F3A/B* G34 genetic alteration. Subtle genetic differences between the two lesions showed that the cervical lesion was not a metastasis from the pelvic tumour. Our analysis also confirmed that these osteoclast-rich tumours were genetically distinct from cGCTB.

The possibility exists that these OC-rich lesions represent an extreme manifestation of PDB. A “true” neoplasm would be expected to harbour a somatic driver genetic alteration, which could not be identified in any of the samples analysed. As such, these masses were more consistent with “tumour-like” lesions. The cause of their large size is unclear, but abnormal paracrine stimuli have been shown to play a key role in the initiation and growth of cGCTB, as recently reported by Cottone et al. [5], and represents a potential contributing factor.

From a therapeutic perspective, the clinical management of cGCT in long bones is curettage or excision. However, when a lesion is inoperable, or where resection would be associated with major morbidity, particularly in the pelvis and spine, downstaging prior to surgery with bisphosphonates, RANK ligand inhibitors, tumour embolisation or radiation therapy may be employed [6–8]. Clinical management of osteoclast-rich lesions in PDB is less well defined, reflecting their rarity. If a large symptomatic tumour is present, as in our case, surgical removal is likely to be advocated. Antiresorptive agents, which are known to control Paget bone disease [9] through inhibition of osteoclast formation/activity, may provide a valuable option for the treatment of small lesions. To our knowledge, the efficacy of this treatment in Pagetic osteoclast-rich lesions has not been evaluated and would be difficult to prove because the tumours are so rare.

From a diagnostic perspective, the finding of an osteoclast-rich lesion without *H3F3A* mutations should prompt further investigations and PBD should be considered. A diagnosis of PBD has implications for patients and their families, and detection of a germline alteration would allow screening of family members, provision of an early diagnosis and non-invasive monitoring of those harbouring the mutation. Furthermore, antiresorptive agents, such as bisphosphonates and denosumab, already used in the treatment of PBD without osteoclast-rich lesions [9], could potentially avoid the development and progression of such lesions in individuals with germline alterations in *ZNF687* and *PFN1*. Importantly, screening for genetic drivers in PBD is valuable as there remains a significant proportion of patients in whom the genetic basis of the disease is not yet explained [1].

## Data Availability

The sequencing data that support the findings of this study are available from the corresponding author upon request.
